# The influence of static and dynamic warm-up on knee temperature: infrared thermography insights before and after a change of direction exercise

**DOI:** 10.3389/fphys.2024.1393804

**Published:** 2024-08-01

**Authors:** Bruno Trovato, Martina Sortino, Luca Petrigna, Federico Roggio, Giuseppe Musumeci

**Affiliations:** ^1^ Department of Biomedical and Biotechnological Sciences, Section of Anatomy, Histology and Movement Science, School of Medicine, University of Catania, Catania, Italy; ^2^ Research Center on Motor Activities (CRAM), University of Catania, Catania, Italy; ^3^ Department of Biology, Sbarro Institute for Cancer Research and Molecular Medicine, College of Science and Technology, Temple University, Philadelphia, United States

**Keywords:** infrared thermography, thermoregulation, stretching, warm-up, knee, exercise

## Abstract

**Introduction:**

Infrared thermography is gaining attention in the field of sports medicine and performance. This study investigated the effects of static and dynamic warm-ups and a 90° change of direction (COD) exercise on the thermal response of the knee.

**Methods:**

Thermograms were collected using the FlIR E54 Imaging Camera from 85 healthy young adults, 46 men and 39 women, aged 20–31 years. The participants were divided in two groups, static and dynamic warm-up. Four thermograms were taken: baseline (T0), warm-up (T1), COD (T2), and rest (T3). Four regions of interest (ROIs) of the knee were analyzed: anterior upper half (AUH), anterior lower half (ALH), posterior upper half (PUH), and posterior lower half (PLH). Mixed ANOVA with the Bonferroni–Holm test and independent *t*-test were used for pairwise comparison and to spot differences between the right and left knees at T1 and T2 and at T0 between men and women, respectively.

**Results:**

The mixed ANOVA was significant for time points (*p*< 0.001) in all the ROIs and for the stretching/temperature interaction with different levels of significance. The *t*-test results for the right and left knees at T1 and T2 were not significant. The temperature in the static warm-up group followed a decrease at T1, a subsequent decrease at T2, and a recovery similar to the baseline at T3, in the ALH in men and women and in the PUH only in men.

**Conclusion:**

Static stretching was more suitable for preparing the knee for the COD exercise than the dynamic one in terms of the thermal response.

## 1 Introduction

Warm-up is a fundamental moment in which both recreational and professional athletes prepare themselves physically and psychologically to perform intense physical activity. It has been observed that various types of warm-ups can reduce the risk of injuries in the lower limbs (Herman et al., 2012; Behm et al., 2016). The implementation of stretching exercises was explored in the past, with controversial findings, describing whether static or dynamic stretching was useful or not in preparing the muscles for subsequent activity with the potential to reduce the incidence of injuries (Herman et al., 2012; Behm et al., 2016; Opplert and Babault, 2018; Chaabene et al., 2019).

Change of direction (COD) exercises require a high physical demand due to the forces that converge on the different anatomical structures in the area of the knee both for the joint (Donelon et al., 2020) and for the muscles (Kekelekis et al., 2022). However, they are key movements to master for athletes to reach a high peak of performance, especially in team sports (Loturco et al., 2022), where performing them with high efficacy is considered an indicator of talent (Scanlan et al., 2021). The demands on the knee, when performing a COD exercise, highly depend on the velocity and angle of the action, with 90 degree COD determining higher braking and propulsive forces than other angles (Schot et al., 1995). Therefore, it is fundamental to choose the right warm-up for athletes before they approach physical activities involving COD exercises, considering that this maneuver has been considered one of the main actions resulting in non-contact injuries of the anterior cruciate ligament in different sports (Waldén et al., 2015; Montgomery et al., 2018).

Nowadays, innovative technologies provide researchers and clinicians new tools to use and new data to analyze, allowing an improvement in the understanding of human physiology both under static (Singla and Veqar, 2014) and dynamic (Roggio et al., 2021) conditions. One of the most interesting and promising techniques in this field is infrared thermography (IRT), which is used in different kinds of evaluations, from clinical medicine to sports and exercise physiology (Hildebrandt et al., 2010; Ring and Ammer, 2012). IRT evaluations are easy to perform, and the technique is non-harmful, highly reproducible (Zaproudina et al., 2008; da Silva et al., 2022), and reliable under different conditions when performed following specific protocols for thermal data acquisition (Moreira et al., 2017). IRT cameras use a sophisticated form of the radiation thermometer to detect heat radiation emitted by the human body within an electromagnetic spectrum that is not visible to the human eye (Costa et al., 2016). By identifying the surface temperature, these cameras provide insights into the blood flow and muscle metabolism, which, in turn, enhance the convection of heat across various regions of the body (del et al., 2017). The outcome measurement evaluated by this technique is the skin temperature (Tsk) distribution of the region of interest (ROI) framed by the camera (Jones and Plassmann, 2002). Studies support the application of IRT in the detection of Tsk changes after different kinds of exercises (Fernandes et al., 2016; Ferreira-Júnior et al., 2021; Alburquerque et al., 2022), as an imaging strategy to reduce the incidence of muscle injuries (Côrte et al., 2019; Gómez-Carmona et al., 2020) and to monitor the fatigue after specific warm-up exercises (Hadžić et al., 2019).

Past studies investigated the effects of static or dynamic stretching on COD performance (Beckett et al., 2009; Wong et al., 2011; Turki et al., 2019) in terms of velocity, power, and biomechanics improvement. To date, the thermal response of the anatomical structures in the area of the knee after a COD exercise, anticipated by a static or dynamic stretching warm-up, was never explored. These structures are fundamental while practicing different athletic movements, from individual to team sports. To optimize the warm-up routines, considering the thermal response of the knee is fundamental to improve the wellness of athletes and help coaches in tailoring to their training interventions. Moreover, this study will provide researchers with further insights into the thermoregulatory response of the knee and new perspectives on how to adjust the warm-up interventions to better suit the physical demands of exercise. Hence, the aim of this study was to investigate the efficacy of static and dynamic stretching warm-ups in preparing the knee for a 90° COD exercise, utilizing IRT. Additionally, the study aimed to analyze the thermal responses in healthy young adults, differentiating between genders and types of stretching.

## 2 Methods

This pre–post-observational study involved 85 healthy young adults (46 men [M] and 39 women [F]) aged 20–31 years and evaluated the Tsk of the knees with IRT at four time points before and after different types of exercises. The study was approved by the Research Center on Motor Activities (CRAM) Scientific Committee (Protocol no. CRAM-020-2021, 20/12/2021) in accordance with the Declaration of Helsinki. Informed consent was obtained from all subjects before participation.

### 2.1 Participants

Participants were recruited voluntarily at the Research Center on Motor Activities (CRAM), University of Catania. The inclusion criteria were age between 18 and 35 years, body mass index (BMI) between 18 and 25 kg/m^2^, and no altered health status. A questionnaire was administered to all participants to collect general information about allergies, medication use, recent surgery, regular menstrual cycle, sports played, and dominant limb. The exclusion criteria were musculoskeletal disorders, history of scoliosis or spine alterations, acute back pain during the previous 4 months, recent surgery, altered menstrual cycle, diabetes, or metabolic syndrome.

### 2.2 Data collection and instruments

The IRT acquisitions were performed according to the TISEM checklist (Moreira et al., 2017) to obtain high-quality thermal images and reduce bias. This checklist allows researchers to perform reliable data acquisition with IRT taking into account extrinsic factors affecting Tsk, like applications of ointments, temperature and humidity, consumption of drugs before the measurement, and positioning of the camera. Thermograms were taken using a FLIR E54 camera (Wilsonville, OR, United States) with a detector resolution of 320 × 240 pixels and thermal sensitivity <0.04°C. A tripod was placed in a room with a set temperature of 24°C ± 2°C and humidity of 50%, 1.5 m away from the subjects to standardize the image acquisition; the sensor’s emissivity was set at 0.98. The process of IRT involves capturing the radiance of an object and then using algorithms to convert it into temperature values, providing information about the temperature of the object’s surface. To ensure accurate results, the participants were asked to wear shorts to not influence the knee temperature; moreover, they rested for 15 min in the evaluation room for acclimatization before the thermogram acquisition. During the imaging, the participants stood upright with their knees to the camera, arms by their sides, in shorts, facing the camera with their patella and after with their popliteal fossa. The thermograms of both the right and left knees were acquired at different time points: baseline (T0), Tsk after warm-up (T1), Tsk after COD exercise (T2), and Tsk after a 20-min rest (T3).

**FIGURE 1:**
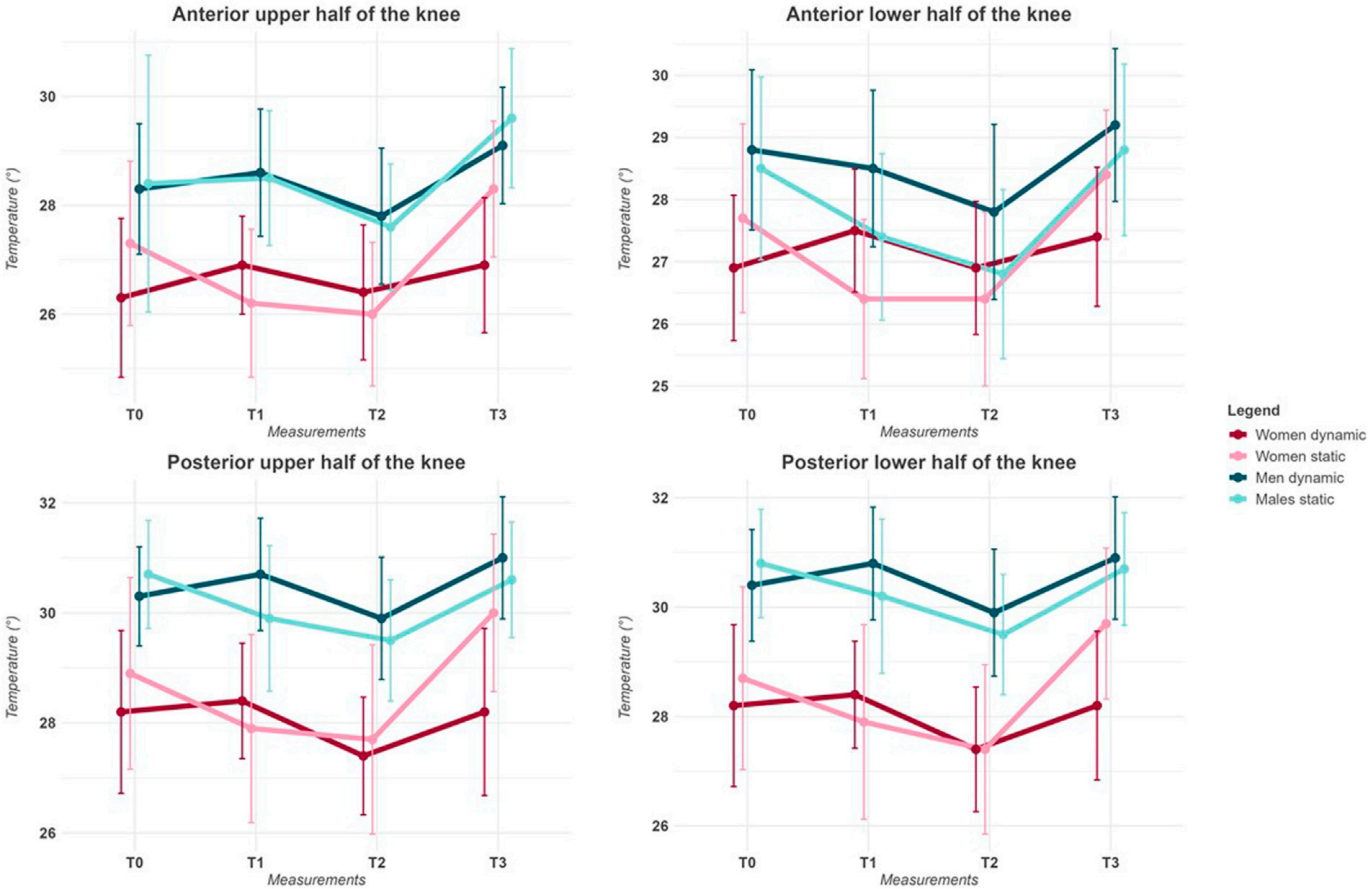
Representation of IRT acquisition and ROI division of the anterior and posterior regions of the knee. The thermograms were edited using FLIR Thermal Studio PRO, version 1.9.38.0.

Using FLIR Thermal Studio PRO software, version 1.9.38.0, we analyzed the thermograms, focusing on two ROIs for the anterior coronal plane and two ROIs for the posterior coronal plane. When an area is delimited on a thermogram, this software application automatically provides the user the average Tsk of the region of interest, representing the average temperature of all the portions selected. Different metrics can be used to evaluate a thermogram other than the mean value; however, the average Tsk was used considering that, to date, it is the most frequently used metric in literature (Perpetuini et al., 2021). For the anterior coronal plane, the ROIs selected were the anterior knee upper half (AUH), comprehending the insertion of the quadriceps and the upper half of the patella, and the anterior knee lower half (ALH), comprehending the origin of the tibialis anterior and the inferior part of the patella. For the posterior coronal plane, the ROIs selected were the posterior knee upper half (PUH), which includes the insertion of the hamstrings and the upper half of the popliteal fossa, and the posterior knee lower half (PLH), including the origin of the gastrocnemius and the lower half of the popliteal fossa. The identification of the anterior and posterior ROIs is presented in Figure 1.

### 2.3 Physical activity protocol

Participants were randomly divided and assigned to two groups, dynamic stretching (26 M and 21 F) and static stretching (20 M and 18 F), and they performed 10 min of warm-up. Then, they performed a 90° COD exercise, focusing the activity on the right knee. The static stretching protocol comprehended 5 min of low-intensity running and 5 min of static stretching of the lower limbs (without reaching the point of discomfort), changing the static position every 30 s. The dynamic stretching protocol comprehended 5 min of low-intensity running and 5 min of dynamic stretching exercise with repeated movements of flexion, extension, adduction, and abduction of the lower limbs. The 90° COD exercise was set up by predisposing a square of 5 m for each section delimited with four cone markers, and the participants had to sprint until they reached the marker and change their direction to reach the subsequent one; the exercise was considered adequately completed when a participant performed five consecutive laps. All the exercises were supervised by an expert trainer.

### 2.4 Statistical analysis

The data analysis was conducted using the R Project for Statistical Computing (Vienna, Austria, v.4.3.2). Data were tested for normality with the Shapiro–Wilk test and equality of variance with Levene’s test. A mixed ANOVA with a *post hoc* Bonferroni–Holm test was used to spot the difference in Tsk in time points and between groups for the type of stretching. A repeated-measures ANOVA was used to spot any significant differences between the temperatures of the right and left knees. The independent *t*-test was used to assess the differences between men and women at T0 and whether differences were present between the temperatures of the right and left knees at T1 and T2.

## 3 Results

The characteristics of the participants, in terms of means and standard deviations for anthropometrics, BMI, age, and temperature at baseline, are presented in Table 1. The IRT results of all the ROIs examined in both the anterior and posterior coronal planes in means, divided by gender, and type of stretching, for the right knee, are presented in Table 2.

**TABLE 1:** Characteristics of the sample.

	Males	Females	*t*-test
	Mean ± SD	Mean ± SD	
Age (years)	21.7 ± 0.5	22.1 ± 2.7	0.027 *
Height (cm)	177 ± 6.5	162 ± 5.7	<0.001 ***
Weight (kg)	71.8 ± 9.5	58.5 ± 7.0	<0.001 ***
BMI (kg/m^2^)	22.8 ± 2.4	22.2 ± 1.9	0.265
T0 AUH	28.3 ± 1.7	26.7 ± 1.5	<0.001 ***
T0 ALH	28.6 ± 1.3	27.2 ± 1.3	<0.001 ***
T0 PUH	30.5 ± 0.9	28.5 ± 1.6	<0.001 ***
T0 PLH	30.6 ± 1.0	28.4 ± 1.5	<0.001 ***

BMI, body mass index; SD, standard deviation; T0, temperature at baseline; AUH, anterior upper half; ALH, anterior lower half; PUH, posterior upper half; PLH, posterior lower half. *p < 0.05, **p < 0.01, and ***p < 0.001.

**TABLE 2:** Mean temperature of the four ROIs of the right knee at each time point.

	Gender	Stretching	AUH T0	ALH T0	PUH T0	PLH to	AUH T1	ALH T1	PUH T1	PLH T1	AUH T2	ALH T2	PUH T2	PLH T2	AUH T3	ALH T3	PUH T3	PLH T3
Mean ± SD	Males	Static	28.4 ± 2.36	28.5 ± 1.47	30.7 ± 0.98	30.8 ± 0.99	28.5 ± 1.24	27.4 ± 1.34	29.9 ± 1.32	30.2 ± 1.41	27.6 ± 1.16	26.8 ± 1.36	29.5 ± 1.10	29.5 ± 1.10	29.6 ± 1.28	28.8 ± 1.38	30.6 ± 1.05	30.7 ± 1.03
		Dynamic	28.3 ± 1.20	28.8 ± 1.29	30.3 ± 0.90	30.4 ± 1.02	28.6 ± 1.17	28.5 ± 1.26	30.7 ± 1.02	30.8 ± 1.03	27.8 ± 1.25	27.8 ± 1.41	29.9 ± 1.11	29.9 ± 1.16	29.1 ± 1.07	29.2 ± 1.23	31.0 ± 1.11	30.9 ± 1.12
	Females	Static	27.3 ± 1.51	27.7 ± 1.52	28.9 ± 1.74	28.7 ± 1.67	26.2 ± 1.36	26.4 ± 1.28	27.9 ± 1.71	27.9 ± 1.78	26.0 ± 1.32	26.4 ± 1.40	27.7 ± 1.72	27.4 ± 1.55	28.3 ± 1.25	28.4 ± 1.04	30.0 ± 1.43	29.7 ± 1.38
		Dynamic	26.3 ± 1.46	26.9 ± 1.17	28.2 ± 1.48	28.2 ± 1.48	26.9 ± 0.90	27.5 ± 0.99	28.4 ± 1.05	28.4 ± 0.98	26.4 ± 1.24	26.9 ± 1.07	27.4 ± 1.07	27.4 ± 1.14	26.9 ± 1.24	27.4 ± 1.12	28.2 ± 1.52	28.2 ± 1.36

SD, standard deviation; AUH, anterior upper half; ALH, anterior lower half; PUH, posterior upper half; PLH, posterior lower half; T0, baseline; T1, temperature after warm-up; T2, temperature after COD exercise; T3, temperature after 20 min of rest.

All data respected the assumption of normality and homogeneity in the Shapiro–Wilk test and Levene’s test, respectively. The temperature of all four ROIs analyzed was higher in men at each of the time points T0, T1, T2, and T3 for both the right and left knees. The comparison between the right and left knees showed no significant differences, and the repeated-measure ANOVA results between the right and left knees were not significant. Hence, only the results for the right knee are presented. The general trend of temperature was a slight decrease in temperature at T1, followed by a higher decrease at T2 and a recovery of temperature, near or superior to the baseline at T3. To identify statistical significance, a mixed ANOVA was used to analyze the four ROIs individually and for gender separately. The results of the mixed ANOVA are presented in Table 3.

**TABLE 3:** Mixed ANOVA results of the four regions of interest divided by gender.

Gender	ROI	Stretching	Time points	Stretching: time points
Male	AUH	0.575	<0.001	0.745
	ALH	0.066	<0.001	0.004
	PUH	0.278	<0.001	<0.001
	PLH	0.519	<0.001	0.004
Female	AUH	0.369	<0.001	<0.001
	ALH	0.865	<0.001	<0.001
	PUH	0.167	<0.001	<0.001
	PLH	0.351	<0.001	<0.001

ROI, region of interest; AUH, anterior lower half; ALH, anterior lower half; PUH, posterior upper half; PLH, posterior lower half.

**FIGURE 2:**
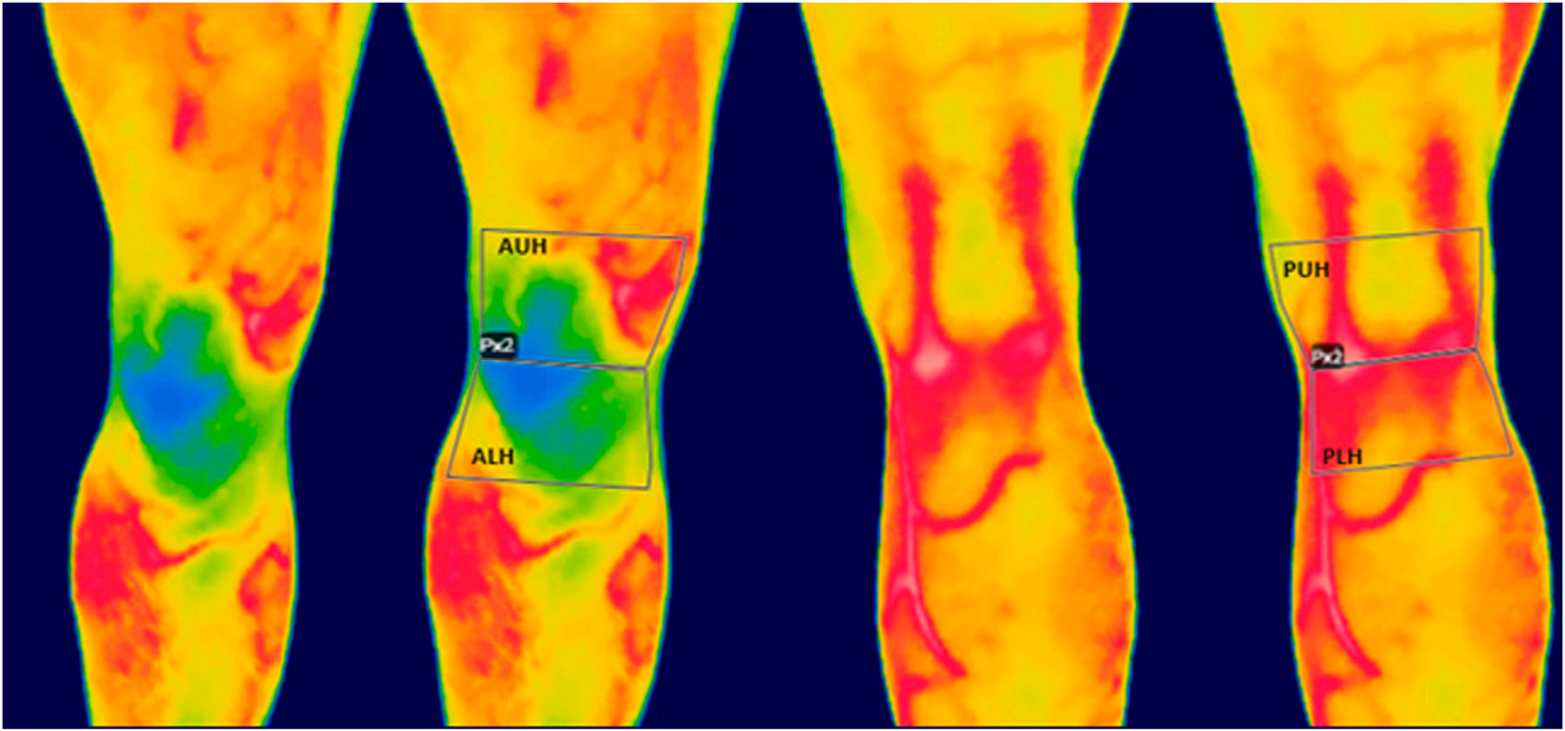
Line plot representing the temperature variations in means at the four time points for each region of interest for men and women divided for stretching.

The type of stretching did not show significant results in the four ROIs examined, with *p*-values >0.05. However, the variable stretching/time points showed significant results in every ROI aside from the AUH. To spot the significance through pairwise comparison, the Bonferroni–Holm *post hoc* test was used, highlighting various significant meaningful comparisons, as shown in Table 4. A significant difference was found between T1 static and T1 dynamic in the ALH for both men and women and the PUH only in men. The dynamic stretching was not significant in all the ROIs examined, while the static stretching elicited a decrease in temperature in the ALH aside from gender and in the PUH only in men. Significant differences were spotted between T0 and T2 in the dynamic group in the ALH for men and in the static group for both men and women in the ALH and PLH and only in men in the PUH. Figure 2 presents the variation in temperature in time points for stretching and gender in the ROIs examined.

**TABLE 4:** Results of the meaningful comparisons of the Bonferroni–Holm test.

Gender	ROI	Stretching	Time points	*p*-value	Gender	ROI	Stretching	Time points	*p*-value
Males	AUH	Static	T0–T1 T0–T2 T2–T3	0.799 0.128 <0.001***	Females	AUH	Static	T0–T1 T0–T2 T2–T3	0.061 0.06 0.001***
		Dynamic	T0–T1 T0–T2 T2–T3	0.564 0.226 <0.001***			Dynamic	T0–T1 T0–T2 T2–T3	0.192 0.747 0.322
		Static: dynamic	T1–T1	0.779			Static: dynamic	T1–T1	0.183
	ALH	Static	T0–T1 T0–T2 T2–T3	0.024* <0.001*** <0.001***		ALH	Static	T0–T1 T0–T2 T2–T3	0.013* 0.013* 0.001***
		Dynamic	T0–T1 T0–T2 T2–T3	0.562 0.022* <0.001***			Dynamic	T0–T1 T0–T2 T2–T3	0.215 0.949 0.237
		Static: dynamic	T1–T1	0.0014***			Static: dynamic	T1–T1	0.032*
	PUH	Static	T0–T1 T0–T2 T2–T3	0.05* 0.004** 0.005**		PUH	Static	T0–T1 T0–T2 T2–T3	0.143 0.071 0.001*
		Dynamic	T0–T1 T0–T2 T2–T3	0.251 0.251 0.002***			Dynamic	T0–T1 T0–T2 T2–T3	0.701 0.2 0.229
		Static: dynamic	T1–T1	0.033*			Static: dynamic	T1–T1	0.453
	PLH	Static	T0–T1 T0–T2 T2–T3	0.148 0.002** 0.007**		PLH	Static	T0–T1 T0–T2 T2–T3	0.148 0.026* 0.001***
		Dynamic	T0–T1 T0–T2 T2–T3	0.292 0.188 0.007**			Dynamic	T0–T1 T0–T2 T2–T3	0.728 0.127 0.116
		Static: dynamic	T1–T1	0.148			Static: dynamic	T1–T1	0.4

ROI, region of interest; AUH, anterior lower half; ALH, anterior lower half; PUH, posterior upper half; PLH, posterior lower half. *p< 0.05; **p< 0.01; and ***p< 0.001.

## 4 Discussion

In this study, the thermal response of the knee after a static or dynamic warm-up and a 90° COD exercise was investigated in a cohort of healthy, young male and female adults. It was found that men presented a higher temperature at the baseline than women. The evaluation of time points showed a general trend of a slight decrease in temperature after warm-up, followed by a higher decrease after the 90° COD exercise and an increase in temperature with a return to baseline or slightly higher at rest. The static stretching warm-up elicited this phenomenon in the ALH in both men and women and in the PUH only in men; instead, the dynamic warm-up did not show significant results in all the ROIs examined. Moreover, the comparison between the static and dynamic groups at T1 showed significant results in the ALH aside from gender and in the PUH in men.

The Tsk is influenced by many factors (Fernández-Cuevas et al., 2015), and differences in temperature at rest between men and women in different body regions have already been investigated in different studies (Chudecka and Lubkowska, 2015; Salamunes et al., 2017). Our study aligns with a previous study that found that men have a higher Tsk than women in the lower limbs (Neves et al., 2017). Women have higher subcutaneous fat percentages than men (Karastergiou et al., 2012), and this characteristic affects the Tsk detection through IRT (Reis et al., 2023). Moreover, the metabolic rate is also higher in men than women (Lazzer et al., 2010), fostering this thermal difference in areas of the body where men typically have more muscle tissue. Marins et al. (2014) also found that there are significant differences in the temperature of the lower limbs between men and women, especially in the calves and ventral and posterior thighs. Thus, it is essential to consider gender when performing IRT acquisition regarding lower limbs.

The general trend of our results showed a slight decrease in Tsk after warm-up exercise and a higher reduction after a more intense COD exercise, followed by an increase in temperature after 20 min of rest, especially in the static group. This temperature trend after acute exercise is in line with different study results (Torii et al., 1992; Fernández-Cuevas et al., 2014; Chudecka et al., 2015; Moreira-Marconi et al., 2019; Lino-Samaniego et al., 2022; Sillero-Quintana et al., 2022), which highlighted a decrease in temperature immediately post-exercise and an increase during the rest period, pursuant to different types of physical activities. When administering incremental exercise, the temperature decreases at the start, with a progressive decrease until exhaustion, and no increase is observed until the end of exercise during rest (Hillen et al., 2020). IRT allows us to detect Tsk, which is the result of the heat transfer of deeper tissue to the upper layers and blood flow (Ammer and Formenti, 2016). The decrease in temperature after acute exercise is caused by the physiological vasodilatation response due to the skin that reflexively contracts as blood is redistributed from non-working tissues to the working skeletal muscle (Charkoudian, 2010). Another physiological reason behind this trend is the start of the thermoregulatory response, particularly sweating, that causes the Tsk to decrease during the first few minutes of exercise compared to thermograms taken at other times; subsequently, during exercise, the Tsk will increase progressively (Fernandes Ade et al., 2014).

We did not find any statistical difference in temperature between the right and left knees. This could be explained by the nature of the exercise that made both legs physically active, considering that temperature follows the same trend in the non-exercised leg (Ferreira et al., 2008).

The physical demand of a sharp 90° COD exercise requires high muscle activation on the biceps femoris and vastus lateralis, while the quadriceps is fundamental for the deceleration phase (Dos’Santos et al., 2018). Moreover, the hamstrings do the essential job of stabilizing the knee to protect it from abnormal movement that can lead to an injury (Neptune et al., 1999; Besier et al., 2003).

Williams et al. (2015) found that static stretching can elicit differences in the quadriceps torque output, analyzing their sample with surface electromyography, and another study (Amiri-Khorasani and Kellis, 2013) found that dynamic stretching can elicit significant differences in electromyographic activity of the rectus femoris and vastus medialis and lateralis.

Conversely, we found that neither static nor dynamic stretching elicited a change in temperature in the AUH. This may be due to the intensity of the warm-up, which was not high enough to prepare one of the biggest muscles in the body for activity in terms of temperature. Generally, static stretching focused on the quadriceps can elicit positive changes in it (Williams et al., 2015); however, the static stretching in our study was focused not only on the quadriceps but also on all the main muscle groups of the legs that contribute to the stabilization of the knee. A lowering of Tsk can be interpreted as a redirection in blood flow from the skin vessels to the muscles (Tanda, 2015), suggesting that muscles might be more ready to perform the exercise. Differently from our results, de Oliveira et al. (2018) observed an increase in muscle temperature of the hamstrings after 180 s of static stretching. However, their study was performed in laboratory settings, while our study focused on testing a stretching warm-up routine closer to that performed before training. Moreover, the authors evaluated all the posterior sections of the thigh, while we analyzed only the insertion of the hamstring close to the knee.

This study has some limitations. First, we examined a sample of people who perform physical activity as a recreational activity, so these findings should be interpreted carefully when comparing with other populations such as athletes. Second, we used the BMI instead of the body fat percentage as an exclusion criterion, which can impact the IRT results. The observed differences in thermal response between men and women suggest that customized warm-up routines might be beneficial for sport practitioners. Thus, incorporating tailored stretching exercises could be more effective in preparing the muscles, potentially reducing injury risk during high-intensity maneuvers like 90° COD exercises. Future studies should evaluate the knee thermal response to stretching in professional athletes and change of direction exercise during a training period and explore possible connections between abnormal thermal response to exercise and the rates of injuries.

## 5 Conclusion

This study presents an exploration into the effects of two distinct stretching warm-ups and a 90° COD exercise on the thermal response of the upper and lower regions of the knee, utilizing IRT. We found that men exhibited a higher Tsk than women. Notably, the static stretching warm-up elicited a significant reduction in Tsk compared to dynamic stretching, particularly in the anterior lower and posterior upper regions of the knee for men and only in the anterior lower region for women. These findings provide valuable practical information for trainers in determining the most suitable stretching warm-up for their athletes tailored to the physical demands required by specific exercises. Coaches can use this information to customize warm-up protocols that optimize knee temperature, preparing their athletes with more precision for subsequent performances, attempting to reduce the risk of potential injuries. Moreover, the results of this study may be useful for researchers investigating the thermal responses of the knee after different kinds of moderate and more intensive exercises.

## Data Availability

The raw data supporting the conclusion of this article will be made available by the authors, without undue reservation.
